# Actual state of “triple therapy” for heart failure patients in eight regions of Japan: An analysis of a nationwide medical claims database

**DOI:** 10.1371/journal.pone.0249711

**Published:** 2021-04-27

**Authors:** Daisuke Abe, Takayuki Inomata

**Affiliations:** 1 Medical Affairs, Upjohn, Pfizer Japan Inc, Shibuya-ku, Tokyo, Japan; 2 Department of Cardiovascular Medicine, Kitasato University Kitasato Institute Hospital, Minato-ku, Tokyo, Japan; International University of Health and Welfare, School of Medicine, JAPAN

## Abstract

**Background:**

This study aimed to collect data on “triple therapy” for heart failure (HF) with angiotensin-converting enzyme inhibitors (or receptor blockers), β-blockers, and mineralocorticoid receptor antagonists in all eight regions of Japan and clarify the reason for the selection of this therapeutic approach.

**Methods and results:**

We used data from April 2017 to March 2018 from the Medical Data Vision database (380 facilities) to analyze factors impacting triple therapy for HF. Among patients who were hospitalized for HF during the study period, 51,933 patients met the inclusion criteria and underwent further analyses. A reference value of 20.45% from Kanto was used to compare the eight Japanese regions.

From the patient cohort, 10,006 (19.27%) patients receiving triple therapy were identified.

The highest and lowest rates of triple therapy were in Chugoku (21.90%) and Shikoku (14.27%), respectively, suggesting regional differences in the use of triple therapy at discharge for patients with HF (P < 0.001). Regression analysis revealed a decrease in the administration of triple therapy for patients with chronic kidney disease (odds ratio [OR], 0.45; 95% confidence interval [CI], 0.43–0.48]; P < 0.001), those aged 75 years and older (OR, 0.46, 95% CI: 0.44–0.49; P < 0.001), those from Shikoku (OR, 0.69; 95% CI, 0.60–0.80; P < 0.001), those with chronic obstructive pulmonary disease (OR, 0.75; 95% CI, 0.68–0.84; P < 0.001), those with anemia (OR, 0.78; 95% CI, 0.62–0.98; P = 0.034), and those from Tohoku (OR, 0.83; 95% CI, 0.75–0.92; P < 0.001).

**Conclusions:**

Future efforts to rectify the regional variance in drug therapy conforming to the guidelines for the treatment of acute and chronic HF will help to extend the healthy lifespans of patients with HF. Further clarification is required to determine instances where triple therapy should be avoided based on patient factors, and appropriate countermeasures should be identified.

## Introduction

Heart failure (HF) is defined as a “clinical syndrome that involves some form of cardiac dysfunction, that is, where the heart experiences an organic or functional abnormality with a breakdown in the ability to compensate its heart pumping function, resulting in dyspnea, malaise, or edema, and consequently lowering exercise tolerance” [1]. Moreover, the increase in patients with HF constitutes a medical and financial burden for society [2]. According to the Japanese Ministry of Health, Labour, and Welfare’s 2016 demographics survey [3], 198,006 deaths in Japan were due to heart disease (15.1%), making it the second leading cause of death in Japan. Among the deaths from heart disease, 73,545 deaths were due to HF; thus, HF remains a disease with a high mortality rate.

To address this situation, Japan passed a “basic law regarding measures against stroke, heart disease, and other cardiovascular diseases in order to lengthen the healthy life expectancy” in December 2018 [4]. Article 11 sets forth: “Prefectural and city governments shall formulate plans for promoting countermeasures against cardiovascular disease in the prefecture/city that are based on the Basic Plan for Promoting Cardiovascular Disease Countermeasures, and that take into account prevention of cardiovascular disease in the prefecture/city, the health of patients with cardiovascular disease, the situation regarding the medical and welfare services provided, and advances in research on cardiovascular disease” [4].

HF is broadly divided into non-ischemic dilated cardiomyopathy and ischemic cardiomyopathy, based on the cause of cardiac dysfunction. In these diseases, the sympathetic nervous system and the renin-angiotensin-aldosterone system are activated, producing progressive left ventricular dilatation and reduced contractility, that is, remodeling, causing death or worsening of HF [5]. Thus, the aim of chronic HF drug therapy is to use drugs to inhibit this neuroendocrine system, thereby reducing left ventricular remodeling and improving lifetime prognosis for patients with HF [1]. During drug therapy for HF, left ventricular ejection fraction (LVEF) < 40%, > 50%, and 40%–49% are defined as HF with reduced ejection fraction (HFrEF), HF with preserved EF (HFpEF), and HF with mid-range LVEF (HFmrEF) or HFpEF borderline, respectively [1]. While patients with a mild reduction in LVEF may present with some degree of systolic dysfunction, their clinical manifestations often overlap with those of HFpEF. However, unlike patients with HFpEF, patients with borderline LVEF may respond well to treatments that have been demonstrated to be effective in the treatment of systolic dysfunction in HFrEF.

Considering the central role of the renin-angiotensin-aldosterone system and the sympathetic nervous system in HF with reduced HFrEF, angiotensin-converting enzyme inhibitors (ACEIs) [6,7] or angiotensin II receptor blockers (ARBs) [8,9], β-blockers [10,11], and mineralocorticoid receptor antagonists (MRAs) [12,13] are administered for such patients; these are Class I, evidence level A recommended drugs that improve the life prognosis of HFrEF patients [1]. The administration of these Class I recommended drugs for patients with HF is therefore expected to lead to better prognoses for patients with HF, especially in HFrEF, and the combination therapy of ACEIs, ARBs, and MRAs is recommended by professional guidelines [14,15]. Despite evidence of the benefit of triple therapy in HFrEF patients, the role of β-blockers in treating HFpEF (caused by hypertension, arrhythmias such as atrial fibrillation, coronary heart disease, diabetes mellitus, and dyslipidemia) is controversial [16]. Similarly, for ACEIs/ARBs and MRAs, no prospective interventional studies on drugs for the treatment of HFpEF have demonstrated a clear reduction in the risk of death or clinical events. Currently, patients with HFpEF are recommended to undergo treatment to control both the causative and comorbid conditions and to manage HF symptoms. According to a survey by the Japanese Circulation Society [17], the median prescription rate of ACEIs or ARBs for hospitalized patients with HF at 610 facilities was 41.0% (interquartile range [IQR]: 31.7%–49.2%), while the median prescription rate for β-blockers was 38.1% (IQR: 27.8%–47.6%). This suggests that the rate of Class I recommended drugs for patients with HF varies between hospitals where the Diagnosis Procedure Combination (DPC) [18] system has been introduced. However, to date, no research has examined whether this variance depends on the region of residence. Investigating and presenting any variances related to Class I recommended drugs between regions where patients with HF reside, or facilities where they receive treatment could help rectify such variances in drug therapy between regions of residence, or between treating facilities, and contribute to extending the lifespan of patients with HF.

Using the Medical Data Vision (MDV) database, this study aimed to collect data on the use of ACEIs or ARBs, β-blockers, and MRAs (triple therapy) that have Class I recommendations for patients with HF, in all eight regions of Japan, as well as to clarify the reason for selection of this therapeutic approach for HF. Additionally, we analyzed factors affecting triple therapy.

## Methods

Data from the MDV database (380 facilities running DPC) from April 2017 to March 2018 were used to analyze the medical history of patients to determine whether hospitalization due to HF took place in the previous year.

### Primary endpoint

The primary endpoint was to obtain data regarding drug therapies and evaluate the factors impacting triple therapy.

### Subjects

The International Statistical Classification of Diseases and Related Health Problems (“ICD-10”) is a classification created by the World Health Organization (WHO) based on the WHO Charter for the purpose of systematic recording, analysis, interpretation, and comparison of mortality and disease data gathered at different time points from different countries and regions [18]. The inclusion criteria for the present study were as follows: Hospitalization as a patient with an injury or illness with the greatest investment in medical resources; and a main injury or illness name, or name of injury or illness prompting hospitalization categorized as I50.0, I50.1, or I50.9 in the ICD-10. The details regarding the ICD-10 are as follows:

- I50.0: Congestive heart failure, congestive heart disease, right ventricular failure (secondary to left heart failure)
- I50.1: Left ventricular failure, cardiac asthma, left heart failure, pulmonary edema, heart disease, NOS, or heart failure
- I50.9: Heart failure, details unknown, heart failure, or myocardial insufficiency NOS

Among hospitalized patients within the aforementioned categories, patients who did not undergo cardiovascular surgery, percutaneous coronary angioplasty, or percutaneous coronary intervention (PCI), who survived until discharge, and had no readmission planned in advance, were included in the database.

The exclusion criteria included patients with a discharge date after March 31, 2018, or those aged < 15 years at the time of hospitalization.

This study was conducted in accordance with the “Declaration of Helsinki” and with the approval of the Kitasato University Laboratory Hospital Ethics Committee (Study number: 19036). since the study does not contain data subject to privacy laws, there was no requirement to obtain informed consent from the patients by Pfizer. All data were fully anonymized before we accessed them during the period from April 2016 to March 2018.

### Statistical analysis

An inter-group comparison was made between two groups: The Class I-recommended drug triple therapy group and the control group (hereafter referred to as “the other group”). A two-group comparison of continuous quantities was performed using the t-test; comparison of the median was performed using the Wilcoxon rank-sum test; and categorical comparison between the two groups was performed using the chi-square test. For factors that impact triple therapy, the OR using logistic regression analysis was calculated using a stepwise method. Data handling and statistical analyses were performed using SAS version 9.4 (SAS Institute Inc., Cary, NC, USA).

## Results

Among the patients with HF who were hospitalized for HF between April 2017 and March 2018 and survived until the discharge date of March 31, 2018, 54,702 patients met the inclusion criteria.

Ultimately, a total of 51,933 cases were included in the study, after excluding 2,586 patients whose hospital stay surpassed March 31, 2018, and 183 patients who were aged < 15 years at the time of hospitalization.

**Fig 1:**
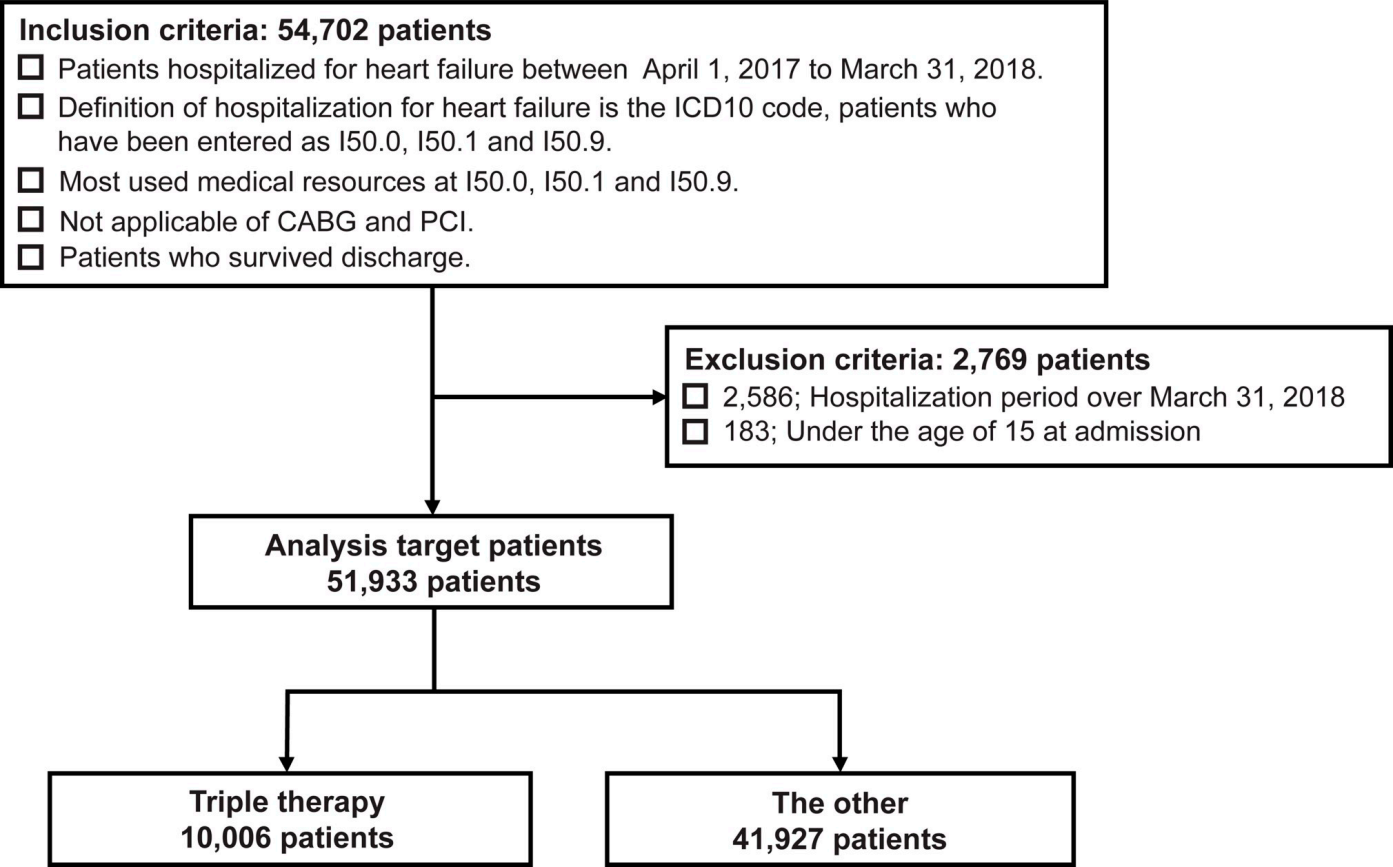
Selection of patients with HF. The triple therapy group received angiotensin-converting enzyme inhibitors (or angiotensin II receptor blockers), β-blockers, and mineralocorticoid receptor antagonists. The other group consisted of the remaining patients who did not receive triple therapy. ICD10: The 10th revision of the International Classification of Diseases and related health problems, CABG: Coronary artery bypass grafting, PCI: Percutaneous coronary intervention.

Patients who were administered triple therapy (ACEIs or ARBs, β-blockers, and MRAs) at discharge were classified under the “triple therapy group” and the remaining patients were classified under the “other group.” There was a total of 10,006 cases in the triple therapy group and 41,927 cases in the other group (Fig 1).

### Characteristics of study subjects

Tables 1 and S1 (Supporting information) show the characteristics of the study subjects. Since patients aged 100 years and older are all entered into the MDV medical database as being 100 years of age, the median value was used as a representative value for age distributions. The median age at hospitalization for the total cohort was 82-years-old, and 73.18% of patients were aged 75 years or older. A total of 10,006 patients received triple therapy. Among the 41,927 patients in the Other group, 18,670 patients were prescribed ACEIs/ARBs, 20,262 patients were prescribed beta-blockers, and 12,889 patients were prescribed MRAs (Table 1).

**Table 1:** Characteristics of patients receiving triple therapy.

Patient characteristics		Total Cohort	Triple therapy	The other	P-value
Total	[n (%)]	51,933	10,006 (19.27)	41,927 (80.73)	
Admission age	Median (IQR)	82 (74–88)	78 (68–85)	83 (75–89)	< 0.001
	Aged 75 years and older [n (%)]	38,003 (73.18)	5,995 (59.91)	32,008 (76.34)	< 0.001
Male	[n (%)]	27,153 (52.28)	5,805 (58.02)	21,348 (50.92)	< 0.001
BMI	Mean ± SD	22.71 ± 5.18	23.61 ± 5.25	22.49 ± 5.14	< 0.001
	Missing [n (%)]	3,389 (6.53)	485 (4.85)	2,904 (6.93)	
Length of stay in hospital days (mean ± SD)		21.09 ± 20.01	19.48 ± 17.09	21.48 ± 20.62	< 0.001
Smoking status					< 0.001
	Smoking [n (%)]	13,285 (25.58)	3,132 (31.30)	10,153 (24.22)	
	No Smoking History [n (%)]	32,752 (63.07)	5,747 (57.44)	27,005 (64.41)	
	Missing [n (%)]	5,896 (11.35)	1,127 (11.26)	4,769 (11.37)	
Medical history					
	Hypertension [n (%)]	42,541 (81.92)	8,785 (87.80)	33,756 (80.51)	< 0.001
	Diabetes mellitus [n (%)]	11,114 (21.40)	2,271 (22.70)	8,843 (21.09)	< 0.001
	Dyslipidemia [n (%)]	24,816 (47.78)	5,349 (53.46)	19,467 (46.43)	< 0.001
	Anemia [n (%)]	660 (1.27)	94 (0.94)	566 (1.35)	0.001
	Atrial fibrillation [n (%)]	24,530 (47.23)	4,961 (49.58)	19,569 (46.67)	< 0.001
	Cerebral diseases [n (%)]	138 (0.27)	24 (0.24)	114 (0.27)	0.576
	Myocardial infarction [n (%)]	14,112 (27.17)	2,818 (28.16)	11,294 (26.94)	0.013
	COPD [n (%)]	3,042 (5.86)	489 (4.89)	2,553 (6.09)	< 0.001
	CKD [n (%)]	14,204 (27.35)	1,742 (17.41)	12,462 (29.72)	< 0.001
Heart failure hospitalization in the previous 180 days [n (%)]		7,420 (14.29)	1,311 (13.10)	6,109 (14.57)	< 0.001
By ambulance to aospital [n (%)]		18,383 (35.40)	3,535 (35.33)	14,848 (35.41)	0.873
Admission of intravenous medications or inhalation					
	hANP [n (%)]	5,866 (11.30)	1,674 (16.73)	4,192 (10.00)	< 0.001
	Diuretic [n (%)]	3,854 (7.42)	904 (9.03)	2,950 (7.04)	< 0.001
	Cardiotonic [n (%)]	136 (0.26)	31 (0.31)	105 (0.25)	0.296
	Oxygen [n (%)]	19,992 (38.50)	4,146 (41.44)	15,846 (37.79)	< 0.001
At discharge medications					
	ACEIs [n (%)]	12,498 (24.07)	5,518 (55.15)	6,980 (16.65)	< 0.001
	ARBs [n (%)]	17,534 (33.76)	5,080 (50.77)	12,454 (29.70)	< 0.001
	ACEIs/ARBs [n (%)]	28,676 (55.22)	10,006 (100.00)	18,670 (44.53)	< 0.001
	β-blockers [n (%)]	30,268 (58.28)	10,006 (100.00)	20,262 (48.33)	< 0.001
	MRAs [n (%)]	22,895 (44.09)	10,006 (100.00)	12,889 (30.74)	< 0.001
	Loop diuretics [n (%)]	42,185 (81.23)	9,315 (93.09)	32,870 (78.40)	< 0.001
	Thiazide [n (%)]	4,255 (8.19)	912 (9.11)	3,343 (7.97)	< 0.001
	Tolvaptan [n (%)]	12,609 (24.28)	3,063 (30.61)	9,546 (22.77)	< 0.001
	Digitalis [n (%)]	3,187 (6.14)	737 (7.37)	2,450 (5.84)	< 0.001
	Nitrate [n (%)]	6,890 (13.27)	1,320 (13.19)	5,570 (13.28)	0.806
	Ca^2+^ channel blockers [n (%)]	20,752 (39.96)	3,781 (37.79)	16,971 (40.48)	< 0.001
	Statins [n (%)]	16,798 (32.35)	4,258 (42.55)	12,540 (29.91)	< 0.001

Data are presented as number of cases and relative percentage of the group’s total in parentheses, except for admission age presented as median with interquartile range in parentheses; BMI and days of hospital stay are presented as mean ± standard deviation. The triple therapy group received angiotensin-converting enzyme inhibitors (or angiotensin II receptor blockers), β-blockers, and mineralocorticoid receptor antagonists. IQR: Interquartile range, BMI: Body mass index, COPD: Chronic obstructive pulmonary disease, CKD: Chronic kidney disease, hANP: Human atrial natriuretic peptide, ACEIs: Angiotensin converting enzyme inhibitors, ARBs: Angiotensin II receptor blockers, ACEIs/ARBs: Angiotensin converting enzyme inhibitors, angiotensin II receptor blockers, or both, MRAs: Mineralocorticoid receptor antagonists treatment, Ca^2+^: Calcium.

In terms of medical history, hypertension was the most frequent comorbidity (81.92%), followed by dyslipidemia (47.78%), atrial fibrillation (47.23%), chronic kidney disease (CKD) (27.35%), myocardial infarction (27.17%), and diabetes mellitus (21.40%). The drug therapies at discharge were as follows: ACEIs, 24.07%; ARBs, 33.76%; ACEIs/ARBs, 55.22%; β-blockers, 58.28%; and MRAs, 44.09%.

Among the cohort, 19.27% (10,006 cases) of patients had ACEIs/ARBs, β-blockers, and MRAs (triple therapy) (Table 1). The median age at the time of hospitalization in the triple therapy group was 78 years, versus 83 years in the other group. Patients aged 75 years and older accounted for 59.91% of the triple therapy group and 76.34% of the other group, representing a higher proportion of subjects aged 75 years and older in the other group. Males accounted for 58.02% of the triple therapy group and 50.92% of the other group, representing a higher number of males in the triple therapy group (P < 0.001 for age at discharge, proportion of patients aged 75 years and older, and proportion of mean, each).

There were only four variables for which no significant differences were verified between the two groups: history of cerebrovascular disease (triple therapy group: 24 [0.24%]; other group, 114 [0.27%]; P = 0.576); transported by ambulance to the hospital (triple therapy group: 3,535 (35.33%), other group: 14,848 (35.41%); P = 0.873); administration of cardiotonic (triple therapy group: 31 [0.31%], other group: 105 [0.25%]; P = 0.296); and use of nitrate at discharge (triple therapy group: 1,320 [13.19%], other group: 5,570 [13.28%]; P = 0.806).

### Triple therapy ratio at discharge for the eight regions in Japan

Table 2 shows the use of triple therapy at discharge in facilities running DPC in eight primary regions for the 51,933 cases in our study. The ratio of triple therapy was as follows: Hokkaido, 19.53%; Tohoku, 17.54%; Kanto, 20.45%; Chubu, 18.18%; Kinki, 18.38%; Chugoku, 21.90%; Shikoku, 14.27%; and Kyushu, 20.19%. These findings suggest that there are regional differences in the use of ACE inhibitors, ARBs, β-blockers, MRAs, and triple therapy at discharge for patients with HF (P < 0.001 for each comparison). The rate of MRAs, which was added in the 2017 revised Guidelines for Diagnosis and Treatment of Acute and Chronic HF [1], with Class I evidence level A for HFrEF patients, similar to ACE inhibitors, ARBs, and β-blockers, was 45% or higher in Kanto (45.85%), Chugoku (45.33%), and Kyushu (45.72%), but lower in Tohoku (40.96%), Kinki (40.84%), and Shikoku (40.90%).

**Table 2:** Drug therapy rate of patients with HF in eight major regions in Japan.

Eight main areas	Total (%)	Triple therapy (%)	P-value	ACEIs (%)	P-value	ARBs (%)	P-value	ACEIs/ARBs (%)	P-value	β-Blockers (%)	P-value	MRAs (%)	P-value
Hokkaido	2,002 (3.85)	391 (19.53)	P < 0.001	441 (22.03)	P < 0.001	781 (39.01)	P < 0.001	1,151 (57.49)	P < 0.001	1,203 (60.09)	P < 0.001	883 (44.11)	P < 0.001
Tohoku	3,403 (6.55)	597 (17.54)		913 (26.83)		1,080 (31.74)		1,892 (55.60)		1,903 (55.92)		1,394 (40.96)	
Kanto	12,154 (23.40)	2,485 (20.45)		2,749 (22.62)		4,160 (34.23)		6,641 (54.64)		7,325 (60.27)		5,573 (45.85)	
Chubu	10,024 (19.30)	1,822 (18.18)		2,349 (23.43)		3,203 (31.95)		5,300 (52.87)		5,558 (55.45)		4,500 (44.89)	
Kinki	9,964 (19.19)	1,831 (18.38)		2,247 (22.55)		3,463 (34.76)		5,475 (54.95)		5,990 (60.12)		4,069 (40.84)	
Chugoku	4,558 (8.78)	998 (21.90)		1,296 (28.43)		1,466 (32.16)		2,629 (57.68)		2,846 (62.44)		2,066 (45.33)	
Shikoku	1,731 (3.33)	247 (14.27)		372 (21.49)		517 (29.87)		854 (49.34)		870 (50.26)		708 (40.90)	
Kyushu	8,097 (15.59)	1,635 (20.19)		2,131 (26.32)		2,864 (35.37)		4,734 (58.47)		4,573 (56.48)		3,702 (45.72)	

Data are presented as number of cases and relative percentage of the group’s total in parentheses. The P-value of group comparison using the chi-square test is indicated for each drug therapy. The triple therapy group received angiotensin-converting enzyme inhibitors (or angiotensin II receptor blockers), β-blockers, and mineralocorticoid receptor antagonist. ACEIs: Angiotensin converting enzyme inhibitors, ARBs: Angiotensin II receptor blockers, MRAs: Mineralocorticoid receptor antagonists.

### Analysis of factors impacting triple therapy

Factors impacting triple therapy were analyzed by multivariate logistic regression analysis using the following 25 variables: sex; aged 75 years and older; previous medical history (hypertension, diabetes mellitus, dyslipidemia, anemia, atrial fibrillation, cerebral diseases, myocardial infarction, chronic obstructive pulmonary disease [COPD], and CKD); HF hospitalization in the last 180 days; transport by ambulance to the hospital; administration of intravenous medications or via inhalation (human atrial natriuretic polypeptide [hANP], diuretic, cardiotonic, and oxygen); medications at discharge (loop diuretics, thiazide, tolvaptan, digitalis, nitrate, calcium channel blockers, and statins); and eight geographical regions (Hokkaido, Tohoku, Chubu, Kinki, Chugoku, Shikoku, Kyushu, and Kanto as a reference).

Table 3 shows the results for 22 factors selected by the stepwise method. The results indicate that at discharge, there is a relationship between decreased triple therapy for patients with HF with CKD (odds ratio [OR], 0.45; 95% confidence interval [CI], 0.43–0.48; P < 0.001), aged 75 years and older (OR, 0.46; 95% CI, 0.44–0.49; P < 0.001), Shikoku (OR, 0.69; 95% CI, 0.60–0.80; P < 0.001), COPD (OR, 0.75; 95% CI, 0.68–0.84; P < 0.001), anemia (OR, 0.78; 95% CI, 0.62–0.98; P = 0.034), from Tohoku (OR, 0.83; 95% CI, 0.75–0.92; P < 0.001), having had heart failure hospitalization in the previous 180 days (OR, 0.86; 95% CI, 0.81–0.92; P < 0.001), taking a Ca^2+^ channel blocker (OR, 0.89; 95% CI, 0.84–0.93; P < 0.001), and from Chubu (OR, 0.92; 95% CI, 0.85–0.98; P = 0.014). Conversely, there is a relationship between increased triple therapy for patients with HF with loop diuretic (OR, 3.46; 95% CI, 3.19–3.76; P < 0.001), hypertension (OR, 1.81; 95% CI, 1.69–1.93; P < 0.001), use of statins (OR, 1.55; 95% CI, 1.48–1.63; P < 0.001), intravenous medication of hANP on admission (OR, 1.52; 95% CI, 1.42–1.62; P < 0.001), from Tolvaptan (OR, 1.33; 95% CI, 1.26–1.40; P < 0.001), from Chugoku (OR, 1.17; 95% CI, 1.07–1.28; P < 0.001), being male (OR, 1.15; 95% CI, 1.10–1.21; P < 0.001), from Kyushu (OR, 1.12; 95% CI, 1.04–1.21; P = 0.003), arriving by ambulance to hospital (OR, 1.09; 95% CI, 1.04–1.14; P < 0.001), taking thiazide (OR, 1.09; 95% CI, 1.00–1.18; P = 0.043), and atrial fibrillation (OR, 1.08; 95% CI, 1.03–1.13; P = 0.001). A reference value of 20.45% from Kanto was used to compare the eight regions.

**Table 3:** Associations between patient characteristics and triple therapy.

	Adjusted OR	95% CI	P-value
CKD	0.45	0.43–0.48	P < 0.001
Aged 75 years and older	0.46	0.44–0.49	P < 0.001
Shikoku*	0.69	0.60–0.80	P < 0.001
COPD	0.75	0.68–0.84	P < 0.001
Anemia	0.78	0.62–0.98	0.034
Tohoku*	0.83	0.75–0.92	P < 0.001
Heart failure hospitalization in the previous 180 days	0.86	0.81–0.92	P < 0.001
Ca^2+^ channel blocker	0.89	0.84–0.93	P < 0.001
Chubu*	0.92	0.85–0.98	0.014
Kinki*	0.96	0.89–1.03	0.208
Hokkaido*	0.96	0.85–1.09	0.543
Atrial fibrillation	1.08	1.03–1.13	0.001
Thiazide	1.09	1.00–1.18	0.043
By ambulance to hospital	1.09	1.04–1.14	P < 0.001
Kyushu*	1.12	1.04–1.21	0.003
Male	1.15	1.10–1.21	P < 0.001
Chugoku*	1.17	1.07–1.28	P < 0.001
Tolvaptan	1.33	1.26–1.40	P < 0.001
Admission intravenous medication of hANP	1.52	1.42–1.62	P < 0.001
Statin	1.55	1.48–1.63	P < 0.001
Hypertension	1.81	1.69–1.93	P < 0.001
Loop diuretic	3.46	3.19–3.76	P < 0.001

*Reference from Kanto of 20.45%.

ORs and 95%CIs are shown. COPD: Chronic obstructive pulmonary disease, CKD: Chronic kidney disease, hANP: Human atrial natriuretic peptide, Ca: Calcium.

Additional analyses indicated that the factors correlated with the disuse of the components of the triple therapy, such as ACEI/ARB, β-blockers, and MRA. Regarding ACEI/ARB (S2 Table), disuse was correlated with age 75 years and older (OR, 0.61; 95% CI, 0.58–0.63; P < 0.001), anemia (OR, 0.66; 95% CI, 0.56–0.77; P < 0.001), CKD (OR, 0.68; 95% CI, 0.65–0.71; P < 0.001), and Shikoku (OR, 0.85; 95%CI, 0.77–0.95; P = 0.004). Disuse of β-blocker (S3 Table) was correlated with age 75 years and older (OR, 0.41; 95%CI, 0.39–0.43; P < 0.001), Shikoku (OR, 0.70; 95%CI, 0.63–0.78; P < 0.001), COPD (OR, 0.78; 95%CI, 0.72–0.85; P < 0.001), Tohoku (OR, 0.81; 95%CI, 0.75–0.88; P < 0.001), and Chubu (OR, 0.87; 95%CI, 0.82–0.92; P < 0.001). The disuse of MRA (S4 Table) was correlated with CKD (OR, 0.43; 95%CI, 0.41–0.45; P < 0.001), Ca^2+^ channel blocker (OR, 0.69; 95%CI, 0.67–0.72; P < 0.001), Tohoku (OR, 0.75; 95%CI, 0.69–0.81; P < 0.001), aged 75 years and older (OR, 0.77; 95%CI, 0.74–0.81; P < 0.001), Shikoku (OR, 0.78; 95%CI, 0.70–0.87; P < 0.001), Kinki (OR, 0.83; 95%CI, 0.79–0.88; P < 0.001), and Hokkaido (OR, 0.88; 95%CI, 0.80–0.98; P = 0.014).

## Discussion

In the present study, we analyzed data regarding the rate of triple therapy at discharge for patients with HF in all eight regions of Japan, and clarified the factors influencing the selection of triple therapy. Among the total cohort, 19.27% (10,006) of HF patients had triple therapy at discharge, suggesting that facilities running DPC are not fully providing drug therapy that conforms with the Guidelines for Diagnosis and Treatment of Acute and Chronic HF [1]. However, in the absence of data regarding categorization of the type of HF, this percentage might have not captured the actual prescription rate based on the clinical conditions of the patients. Additionally, drug therapy within the eight regions was confirmed to vary from region-to-region. With the passing of the “basic law regarding measures against stroke, heart disease, and other cardiovascular diseases in order to lengthen healthy life expectancy” efforts to rectify the regional variance in drug therapy conforming with the Guidelines for Diagnosis and Treatment of Acute and Chronic HF [1] would contribute to extending the healthy lifespan of patients with HF.

Multivariate logistic regression analysis showed a relationship with decreased triple therapy for patients with CKD, aged 75 years and older, Shikoku, COPD, anemia, from Tohoku, HF hospitalization in the previous 180 days, using a Ca^2+^ channel blocker, and from Chubu. The median age at hospitalization for the total cohort was 82 years, and the proportion of patients aged 75 years or older was 73.18%. According to a survey by the Japanese Circulation Society [17], the mean age of Japanese patients with HF is 75 years for men and 81 years for women; thus, our analysis of the ages of the subjects in the present study conformed to previous research. According to the Statement on the treatment of elderly patients with heart failure [19], aging and CKD impact the prognosis of patients with HF and are also related to decreased use of triple therapy.

The “Cardiovascular disease measure promotion master plan,” published in October 2020 by the Ministry of Health, Labour and Welfare, Japan, identified HF as one of the three most fatal diseases and specified the goal of extending healthy life expectancy by more than 3 years and reducing age-adjusted mortality from cardiovascular disease by 2040 [20]. Against this backdrop, the appropriate use criteria (AUC) for the management of HF was developed to assist physicians in their decision-making in Japan [21]. The report also mentioned that in Japan, where the population is aging, many elderly patients and those with multiple complications are said to account for only about 20% of the cases that can be covered by the clinical guidelines, a fact that highlighted the limitations of the implementation of the guidelines. However, to change this status quo, the AUC has taken into account factors such as age, kidney function, and heart rate. The 19.27% triple therapy rate reported in our study is roughly in line with what is described in the report.

For patients aged ≥ 75 years, disuse of ACEI/ARB and beta-blockers were noted. There is a critical need for practical guidance in Japan in areas that lack robust and conclusive evidence to disseminate clinically actionable information. Therefore, it seems necessary to use AUC to enable the clinical decision-making process for HF management. In addition, there is a need to clarify the factors affecting elderly patients with HF with CKD, such as whether triple therapy should be avoided due to contraindications, or whether triple therapy should be avoided in regard to the patient’s characteristics, and the appropriate countermeasures that need to be considered. These countermeasures should also include therapeutic drugs for HF with new mechanisms of action, which will also be commercially available in Japan, such as ivabradine [22], sacubitril/valsartan [23], omecamtiv mecarbil [24], and sodium-glucose cotransporter 2 (SGLT2) inhibitors [25].

Interestingly, a reduction in triple therapy was observed by region, predominantly Shikoku, Tohoku, and Chubu. However, it is possible that these regional factors can be corrected. When analyzed for different components of triple therapy, Shikoku was found to be correlated with not using ACEI/ARB, beta-blockers, and MRA.

The results of the present study confirm that the rate of triple therapy at discharge for patients with HF is 19.27% and that this rate varies among the eight regions of Japan. Since we could not identify patients with HFrEF from the study subjects, it is not possible to discuss the drug therapy rate conforming with the Guidelines for Diagnosis and Treatment of Acute and Chronic HF [1]. Considering the heterogeneity of diagnostic criteria and baseline characteristics of different subsets of HF patients, the clinical benefit of combination therapy with HFpEF and HFmrEF is controversial, and further evidence is needed for these patients. However, the present study suggests a probable reason for the selection of drug therapy for patients with HF in hospitals where the DPC system has been introduced. Moreover, this research presents new findings regarding measures that will contribute to extending the healthy lifespans of patients with HF.

### Limitations

The present study is the first to report on data regarding drug usage at discharge for patients with HF in eight regions of Japanese facilities running DPC. However, as the database is for facilities running DPC that have entered into a contract with MDV, there are fewer patients for analysis in the Tohoku and Hokkaido regions compared to the 2018 demographic census. As such, it is possible that some deviations have occurred in the regional analysis. Moreover, the DPC data did not include LVEF values; therefore, it is impossible to classify patients with HF included in the analysis based on HFrEF, HFmrEF, and HFpEF, and an analysis restricted only to the subset of patients with HFrEF, for which triple therapy is recommended, cannot be conducted. Therefore, data collection in our study underestimated the prescription rate of the triple therapy. The etiologies of Japanese patients with HFpEF include hypertension and aging [26]. However, from the DPC database, age-related HFpEF cannot be excluded. In an effort to reduce the heterogeneity, we excluded patients with hypertensive congestive HF and hypertensive HF (ICD coding “I11.0”). We also attempted to minimize bias by examining the impact of the OR rather than emphasizing the percentage. Additionally, the DPC data only allow for single-center tracking, making it impossible to know if discharge was followed by readmission to another hospital, death at home, or the like. Thus, it would be difficult to investigate the impact on prognosis, such as readmission for HF, with DPC data alone, and the present study does not stretch to this area of investigation.

As a supplement, the limitations of DPC-based database research, as reported in previous research [27], are described below.

First, the DPC data did not include important information on the clinical status, laboratory data, and cardiac function at admission (such as blood pressure, serum creatinine levels, and ejection fraction). Consequently, the clinical characteristics of patients hospitalized with worsening HF have not been well described. Second, since we could not combine the DPC database with outpatient claims data, the outpatient data after discharge from the index admission were not surveyed.

In addition, although our study has provided important insights into the correlations and prevalence of the factors associated with triple therapy, the cross-sectional nature of the study limited its scope to establish any causal relationships. These points may represent the disadvantages of the current study compared with other registries and observational studies, which may have influenced the results of our analyses.

## Conclusions

Drug therapy conforming to the Guidelines for Diagnosis and Treatment of Acute and Chronic HF by facilities running DPC in all eight regions of Japan were confirmed to vary regionally. Future efforts to rectify this regional variance in drug therapy conforming to the Guidelines for Diagnosis and Treatment of Acute and Chronic HF would contribute to extending healthy lifespans of patients with HF.

## Supporting information

S1 Table Patient characteristics of the entire cohort. (PDF)

S2 Table Associations between patient characteristics and ACEI/ARB. (PDF)

S3 Table Associations between patient characteristics and β-Blocker. (PDF)

S4 Table Associations between patient characteristics and MRA. (PDF)
