# Supplementary material for: Actual state of “triple therapy” for heart failure patients in eight regions of Japan: An analysis of a nationwide medical claims database
Source: PLoS One. 2021 Apr 27;16(4):e0249711. doi: 10.1371/journal.pone.0249711 (PMC8078795; doi:10.1371/journal.pone.0249711)
Supplement: S1 Table — (PDF) [file pone.0249711.s001.pdf]

S1 Table. Patient characteristics of the entire cohort

Patients characteristics		Total	Hokkaido	Tohoku	Kanto	Chubu	Kinki	Chugoku	Shikoku	Kyushu
Total	[n (%)]	51,933	2,002 (3.85)	3,403 (6.55)	12,154 (23.40)	10,024 (19.30)	9,964 (19.19)	4,558 (8.78)	1,731 (3.33)	8,097 (15.59)
Admission Age	median (IQR)	82 (74-88)	82 (73-88)	83 (74-88)	81 (72-87)	83 (74-88)	82 (74-88)	83 (75-88)	83 (75-89)	83 (75-89)
	Aged 75 years and older [n (%)]	38,003 (73.18)	1,461 (72.98)	2,518 (73.99)	8,340 (68.62)	7,435 (74.17)	7,411 (74.38)	3,423 (75.10)	1,331 (76.89)	6,084 (75.14)
Male	[n (%)]	27,153 (52.28)	1,037 (51.80)	1,743 (51.22)	6,675 (54.92)	5,267 (52.54)	5,136 (51.55)	2,313 (50.75)	884 (51.07)	4,098 (50.61)
BMI	mean±SD	22.71±5.18	23.73±11.31	23.25±5.49	22.73±4.67	22.45±4.56	22.62±5.32	22.55±4.41	22.81±4.20	22.74±4.60
	Missing [n (%)]	3,389 (6.53)	222 (11.09)	388 (11.40)	953 (7.84)	473 (4.72)	668 (6.70)	216 (4.74)	86 (4.97)	383 (4.73)
Length of stay in hospital days (mean±SD)		21.09±20.00	20.53±19.91	23.20±20.51	19.91±18.73	20.62±19.78	20.95±19.40	21.14±20.46	21.37±20.91	22.8±21.96
Smoking state										
	Smoking [n (%)]	13,285 (25.58)	551 (27.52)	718 (21.10)	3,376 (27.78)	2,506 (25.00)	2,352 (23.60)	1,337 (29.33)	469 (27.09)	1,976 (24.40)
	No Smoking History [n (%)]	32,752 (63.07)	1161 (57.99)	2,225 (65.38)	6,931 (57.03)	6,326 (63.11)	6,595 (66.19)	2,982 (65.42)	1,173 (67.76)	5,359 (66.19)
	Missing [n (%)]	5,896 (11.35)	290 (14.49)	460 (13.52)	1,847 (15.20)	1,192 (11.89)	1,017 (10.21)	239 (5.24)	89 (5.14)	762 (9.41)
Medical history										
	Hypertension [n (%)]	42,541 (81.92)	1,611 (80.47)	2,906 (85.40)	10,188 (83.82)	8,194 (81.74)	8,040 (80.69)	3,720 (81.61)	1,376 (79.49)	6,506 (80.35)
	Diabetes Mellitus [n (%)]	11,114 (21.40)	214 (10.69)	779 (22.89)	2,515 (20.69)	2,069 (20.64)	2,184 (21.92)	1,157 (25.38)	466 (26.92)	1,730 (21.37)
	Dyslipidemia [n (%)]	24,816 (47.78)	1,000 (49.95)	1,784 (52.42)	6,059 (49.85)	4,567 (45.56)	4,687 (47.04)	2,293 (50.31)	949 (54.82)	3,477 (42.94)
	Anemia [n (%)]	660 (1.27)	63 (3.15)	38 (1.12)	149 (1.23)	124 (1.24)	93 (0.93)	74 (1.62)	12 (0.69)	107 (1.32)
	Atrial Fibrillation [n (%)]	24,530 (47.23)	1,005 (50.20)	1,903 (55.92)	5,649 (46.48)	4,719 (47.08)	4,492 (45.08)	2,254 (49.45)	716 (41.36)	3,792 (46.83)
	Cerebral Diseases [n (%)]	138 (0.27)	1 (0.05)	15 (0.44)	22 (0.18)	35 (0.35)	24 (0.24)	9 (0.20)	2 (0.12)	30 (0.37)
	Myocardial Infarction [n (%)]	14,112 (27.17)	323 (16.13)	953 (28.00)	3,748 (30.84)	2,822 (28.15)	2,671 (26.81)	1,398 (30.67)	491 (28.37)	1,706 (21.07)
	COPD [n (%)]	3,042 (5.86)	110 (5.49)	217 (6.38)	727 (5.98)	585 (5.84)	593 (5.95)	288 (6.32)	80 (4.62)	442 (5.46)
	CKD [n (%)]	14,204 (27.35)	518 (25.87)	943 (27.71)	3,408 (28.04)	2,736 (27.29)	2,576 (25.85)	1,250 (27.42)	477 (27.56)	2,296 (28.36)
Heart Failure Hospitalization Before 180 days [n (%)]		7,420 (14.29)	271 (13.54)	455 (13.37)	1,689 (13.90)	1,472 (14.68)	1,353 (13.58)	708 (15.53)	259 (14.96)	1,213 (14.98)
By Ambulance to Hospital [n (%)]		18,383 (35.40)	744 (37.16)	1,051 (30.88)	4,751 (39.09)	3,445 (34.37)	3,596 (36.09)	1,538 (33.74)	577 (33.33)	2,681 (33.11)
Admission Intravenous Medications or Inhalation										
	hANP [n (%)]	5,866 (11.30)	251 (12.54)	420 (12.34)	1,499 (12.33)	1,213 (12.10)	977 (9.81)	644 (14.13)	116 (6.70)	746 (9.21)
	Diuretic [n (%)]	3,854 (7.42)	139 (6.94)	291 (8.55)	1,008 (8.29)	538 (5.37)	820 (8.23)	297 (6.52)	229 (13.23)	532 (6.57)
	Cardiotonic [n (%)]	136 (0.26)	8 (0.40)	6 (0.18)	30 (0.25)	32 (0.32)	47 (0.47)	6 (0.13)	1 (0.06)	6 (0.07)
	Oxygen [n (%)]	19,992 (38.50)	708 (35.36)	1,361 (39.99)	5,021 (41.31)	3,991 (39.81)	3,574 (35.87)	1,839 (40.35)	609 (35.18)	2,889 (35.68)
At Discharge Medications										
	ACEIs [n (%)]	12,498 (24.07)	441 (22.03)	913 (26.83)	2,749 (22.62)	2,349 (23.43)	2,247 (22.55)	1,296 (28.43)	372 (21.49)	2,131 (26.32)
	ARBs [n (%)]	17,534 (33.76)	781 (39.01)	1,080 (31.74)	4,160 (34.23)	3,203 (31.95)	3,463 (34.76)	1,466 (32.16)	517 (29.87)	2,864 (35.37)
	ACEIs/ARBs [n (%)]	28,676 (55.22)	1,151 (57.49)	1,892 (55.60)	6,641 (54.64)	5,300 (52.87)	5,475 (54.95)	2,629 (57.68)	854 (49.34)	4,734 (58.47)
	β-Blockers [n (%)]	30,268 (58.28)	1,203 (60.09)	1,903 (55.92)	7,325 (60.27)	5,558 (55.45)	5,990 (60.12)	2,846 (62.44)	870 (50.26)	4,573 (56.48)
	MRAs [n (%)]	22,895 (44.09)	883 (44.11)	1,394 (40.96)	5,573 (45.85)	4,500 (44.89)	4,069 (40.84)	2,066 (45.33)	708 (40.90)	3,702 (45.72)
	Triple therapy [n (%)]	10,006 (19.27)	391 (19.53)	597 (17.54)	2,485 (20.45)	1,822 (18.18)	1,831 (18.38)	998 (21.90)	247 (14.27)	1,635 (20.19)
	Loop Diuretics [n (%)]	42,185 (81.23)	1,636 (81.72)	2,850 (83.75)	9,991 (82.20)	8,144 (81.25)	7,886 (79.14)	3,699 (81.15)	1,466 (84.69)	6,513 (80.44)
	Thiazide [n (%)]	4,255 (8.19)	164 (8.19)	236 (6.94)	958 (7.88)	821 (8.19)	712 (7.15)	410 (9.00)	139 (8.03)	815 (10.07)
	Tolvaptan [n (%)]	12,609 (24.28)	623 (31.12)	1,021 (30.00)	2,491 (20.50)	2,358 (23.52)	2,435 (24.44)	1,414 (31.02)	567 (32.76)	1,700 (21.00)
	Digitalis [n (%)]	3,187 (6.14)	138 (6.89)	269 (7.90)	708 (5.83)	666 (6.64)	630 (6.32)	197 (43.22)	88 (5.08)	491 (6.06)
	Nitrate [n (%)]	6,890 (13.27)	357 (17.83)	494 (14.52)	1,410 (11.60)	1,528 (15.24)	1,215 (12.19)	572 (12.55)	351 (20.28)	963 (11.89)
	Ca Channel Blockers [n (%)]	20,752 (39.96)	844 (42.16)	1,335 (39.23)	4,806 (39.54)	3,758 (37.49)	4,274 (42.89)	1,682 (36.90)	721 (41.65)	3,332 (41.15)
	Statins [n (%)]	16,798 (32.35)	667 (33.32)	1,044 (30.68)	4,199 (34.55)	3,075 (30.68)	3,249 (32.61)	1,411 (30.96)	538 (31.08)	2,615 (32.30)

The composition ratio is rounded to one decimal place, so the total does not necessarily add up to 100%
