# Supplementary material for: Actual state of “triple therapy” for heart failure patients in eight regions of Japan: An analysis of a nationwide medical claims database
Source: PLoS One. 2021 Apr 27;16(4):e0249711. doi: 10.1371/journal.pone.0249711 (PMC8078795; doi:10.1371/journal.pone.0249711)
Supplement: S2 Table — (PDF) [file pone.0249711.s002.pdf]

S2 Table. Associations between patient characteristics and ACEI/ARB

	Adjusted Odds Ratio	95% CI	P Value
Aged 75 years and older	0.61	0.58-0.63	P<0.001
Anemia	0.66	0.56-0.77	P<0.001
CKD	0.68	0.65-0.71	P<0.001
Atrial Fibrillation	0.77	0.74-0.80	P<0.001
Heart Failure Hospitalization Before 180 days	0.79	0.75-0.84	P<0.001
COPD	0.81	0.75-0.88	P<0.001
Shikoku *	0.85	0.77-0.95	0.004
Digitalis	0.88	0.81-0.95	0.001
Dyslipidemia	0.91	0.87-0.95	P<0.001
Myocardial Infarction	0.94	0.90-0.98	0.003
Chubu *	1.01	0.96-1.07	0.675
By Ambulance to Hospital	1.04	1.00-1.08	0.047
Kinki *	1.08	1.02-1.14	0.010
Tolvaptan	1.09	1.04-1.14	P<0.001
Tohoku *	1.12	1.03-1.21	0.008
Hokkaido *	1.19	1.07-1.31	0.001
Male	1.23	1.19-1.28	P<0.001
Chugoku *	1.27	1.18-1.37	P<0.001
Kyushu *	1.30	1.22-1.38	P<0.001
Thiazide	1.40	1.30-1.50	P<0.001
Admission Intravenous Medication of hANP	1.57	1.48-1.67	P<0.001
Loop Diuretic	1.72	1.63-1.80	P<0.001
Statin	1.87	1.78-1.96	P<0.001
Ca Channel Blocker	2.04	1.96-2.12	P<0.001
Hypertension	2.32	2.21-2.44	P<0.001

*: Reference of Kanto 54.64%
