# Supplementary material for: Actual state of “triple therapy” for heart failure patients in eight regions of Japan: An analysis of a nationwide medical claims database
Source: PLoS One. 2021 Apr 27;16(4):e0249711. doi: 10.1371/journal.pone.0249711 (PMC8078795; doi:10.1371/journal.pone.0249711)
Supplement: S3 Table — (PDF) [file pone.0249711.s003.pdf]

S3 Table. Associations between patient characteristics and β-Blocker

	Adjusted Odds Ratio	95% CI	P Value
Aged 75 years and older	0.41	0.39-0.43	P<0.001
Shikoku *	0.70	0.63-0.78	P<0.001
COPD	0.78	0.72-0.85	P<0.001
Tohoku *	0.81	0.75-0.88	P<0.001
Anemia	0.84	0.71-0.99	0.032
Chubu *	0.87	0.82-0.92	P<0.001
Ca Channel Blocker	0.90	0.87-0.94	P<0.001
CKD	0.92	0.88-0.96	P<0.001
Kyushu *	0.95	0.89-1.00	0.068
Inhalation Oxygen	0.96	0.92-1.00	0.039
Hokkaido *	1.03	0.93-1.15	0.519
Nitrate	1.06	1.00-1.12	0.048
By Ambulance to Hospital	1.07	1.03-1.12	P<0.001
Thiazide	1.09	1.02-1.17	0.017
Kinki *	1.11	1.05-1.17	P<0.001
Diabetes Mellitus	1.11	1.06-1.17	P<0.001
Male	1.11	1.07-1.16	P<0.001
Myocardial Infarction	1.15	1.10-1.20	P<0.001
Chugoku *	1.16	1.08-1.25	P<0.001
Tolvaptan	1.32	1.26-1.38	P<0.001
Hypertension	1.32	1.26-1.39	P<0.001
Admission Intravenous Medication of hANP	1.33	1.25-1.41	P<0.001
Atrial Fibrillation	1.74	1.68-1.81	P<0.001
Loop Diuretic	1.89	1.80-1.98	P<0.001
Statin	2.09	2.01-2.18	P<0.001

*: Reference of Kanto 60.27%
