# Supplementary material for: Actual state of “triple therapy” for heart failure patients in eight regions of Japan: An analysis of a nationwide medical claims database
Source: PLoS One. 2021 Apr 27;16(4):e0249711. doi: 10.1371/journal.pone.0249711 (PMC8078795; doi:10.1371/journal.pone.0249711)
Supplement: S4 Table — (PDF) [file pone.0249711.s004.pdf]

S4 Table. Associations between patient characteristics and MRA

	Adjusted Odds Ratio	95% CI	P Value
CKD	0.43	0.41-0.45	P<0.001
Ca Channel Blocker	0.69	0.67-0.72	P<0.001
Tohoku *	0.75	0.69-0.81	P<0.001
Aged 75 years and older	0.77	0.74-0.81	P<0.001
Shikoku *	0.78	0.70-0.87	P<0.001
Kinki *	0.83	0.79-0.88	P<0.001
Hokkaido *	0.88	0.80-0.98	0.014
COPD	0.92	0.85-1.00	0.043
Myocardial Infarction	0.92	0.88-0.96	P<0.001
Diabetes Mellitus	0.94	0.90-0.99	0.010
Male	0.95	0.91-0.99	0.006
Chubu *	0.96	0.91-1.02	0.198
Chugoku *	0.97	0.90-1.04	0.349
Kyushu *	1.05	0.99-1.11	0.139
Thiazide	1.09	1.01-1.16	0.019
Intravenous Diuretic	1.12	1.05-1.20	0.001
Statin	1.12	1.08-1.17	P<0.001
Hypertension	1.17	1.12-1.23	P<0.001
Admission Intravenous Medication of hANP	1.22	1.15-1.30	P<0.001
Atrial Fibrillation	1.23	1.19-1.28	P<0.001
Digitalis	1.39	1.28-1.50	P<0.001
Tolvaptan	1.43	1.37-1.49	P<0.001
Loop Diuretic	4.49	4.23-4.76	P<0.001

*: Reference of Kanto 45.85%
